# Defects in the Mitochondrial Genome of Dogs with Recurrent Tumours

**DOI:** 10.3390/ijms252413414

**Published:** 2024-12-14

**Authors:** Krzysztof Kowal, Kaja Ziółkowska-Twarowska, Angelika Tkaczyk-Wlizło, Ludmiła Grzybowska-Szatkowska, Brygida Ślaska

**Affiliations:** 1 Institute of Biological Bases of Animal Production, University of Life Sciences in Lublin, Akademicka 13 St., 20-950 Lublin, Poland; krzysztof.kowal@up.lublin.pl (K.K.); kaja.ziolkowska@up.lublin.pl (K.Z.-T.); angelika.tkaczyk@up.lublin.pl (A.T.-W.); 2 Department of Radiotherapy, Medical University of Lublin, Chodźki 7, 20-093 Lublin, Poland; ludmila.grzybowska-szatkowska@umlub.pl

**Keywords:** Oxford Nanopore Sequencing, mtDNA, dog, cancers

## Abstract

This study presents a comprehensive analysis of mitochondrial DNA (mtDNA) variations in dogs diagnosed with primary and recurrent tumours, employing Oxford Nanopore Technologies (ONT) for sequencing. Our investigation focused on mtDNA extracted from blood and tumour tissues of three dogs, aiming to pinpoint polymorphisms, mutations, and heteroplasmy levels that could influence mitochondrial function in cancer pathogenesis. Notably, we observed the presence of mutations in the D-loop region, especially in the VNTR region, which may be crucial for mitochondrial replication, transcription, and genome stability, suggesting its potential role in cancer progression. The study is pioneering in its use of long-read sequencing to explore the mutational landscape of mtDNA in canine tumours, revealing that while the overall mutational load did not differ between primary and recurrent tumours, specific changes in m.16168A/G, m.16188G/A, and m.16298A/G are linked with tumour tissues. Interestingly, the heteroplasmy outside the D-loop region was not specific to tumour tissues and did not provoke any malignant damage in protein-coding sequences, which in turn may be a tolerant effect of the reactive oxygen species (ROS) cellular stress mechanism.

## 1. Introduction

The knowledge of molecular disorders occurring in the genetic material of cancer cells is mainly related to nuclear DNA (nDNA). However, there is a growing number of reports in which mitochondrial DNA (mtDNA) damage is shown to be important in the neoplastic process [1]. Mitochondria, which have their transcription machineries, play an important role in the cell control of nuclear functions by production of reactive oxygen species (ROS), modulation of calcium levels, and flow control of small molecule metabolites or by regulation of apoptosis [2]. It is, therefore, not surprising that mutations in the mitochondrial genetic material may disrupt cellular homeostasis [3]. Some tissue types give rise to cancers millions of times more often than other tissue types. Although it has been recognized for more than a century, this issue has never been explained [4].

Oxford Nanopore Technologies (ONT), with their distinct ability to sequence long strands of DNA and RNA in real-time, have revolutionized various aspects of genetic research, including the analysis of mitochondrial DNA [5]. Unlike traditional sequencing methods that often require amplification and may struggle with repetitive sequences, the Oxford Nanopore approach facilitates direct real-time analysis of long DNA molecules, providing comprehensive insights into genomic structures and variations [6]. The application of Oxford Nanopore Technologies in mitochondrial DNA analysis is particularly noteworthy. Mitochondrial DNA, with its unique characteristics such as maternal inheritance, high copy number, and frequent mutations, plays a crucial role in understanding human evolution, genetic diseases, and population genetics [7]. The ability of the technology to sequence entire mitochondrial genomes quickly and accurately ensures a more detailed understanding of mtDNA variations and their implications. This is especially important in studies of cancers, where precise identification of mutations is crucial for diagnosis and treatment [8].

In mitochondrial diseases, many mutations affect encoding genes (i.e., *ND5*, *ND4*, *ND1*, and *COX1*), but a region that has been most frequently indicated to have the greatest numbers of polymorphisms and somatic mutations in cancer cells is the D-loop region [9]. This region is a non-coding sequence responsible for genome replication and gene transcription. The available data suggest that mutations in this region may impair the function of the electron transport chain, thereby leading to the generation of increased levels of reactive oxygen species, which damage the DNA structure. Additionally, excess levels of reactive oxygen species can lead to nuclear DNA damage and, consequently, to tumour development [10,11] To date, the exact mechanism of mtDNA mutations in the carcinogenesis process has not been fully elucidated [12]. However, as reported by Kowal et al. (2019), mutations and polymorphisms can affect mitochondrial functions and may be a result of cell adaptation to changes in the environment occurring during carcinogenesis [13].

In the present study, the main aim was to analyse the whole mitochondrial DNA genome isolated from blood, samples of primordial tumours, and tumour recurrences. The authors determined polymorphisms, mutations, and heteroplasmy of mtDNA and their impact on tRNA and protein structure and functions. The changes present in total mtDNA in malignant cancers led to the determination of whether mitochondrial DNA alterations were linked with the carcinogenesis process. This is the first study identifying changes in total mtDNA and their impact on protein and tRNA structure and function in dogs with primordial and recurrent tumours with the use of the Oxford Nanopore Sequencing Technology.

## 2. Results

We analysed 14 mitochondrial sequences obtained from three dogs with different types of cancers. The haplotype was identified for each sample. Table 1 presents haplogroups and haplotypes found in each sample. In dogs A and B, the haplotype was not variable among the analysed sequences, whereas the haplotypes of dog C were different in some samples.

**Figure 1:**
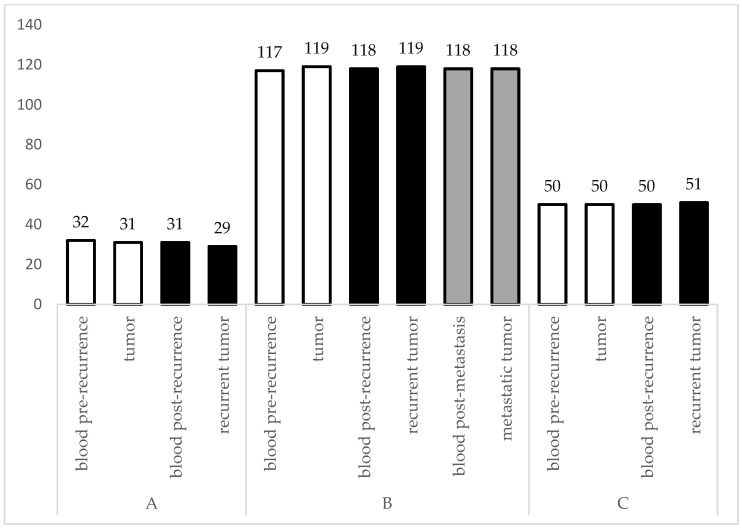
Number of SNPs and indels observed in analysed samples excluding the VNTR region of the D-loop. White bars represent pre-recurrence tissues (primordial tumour and blood samples), whereas black bars represent post-recurrence tissues. Grey bars represent post-metastatic tissues.

We counted the number of variants in each sample (Figure 1). The results are presented in Figure 1. The highest number of SNPs and indels were found in the samples from the crossbreed dog. We observed differences between tumour tissues and blood. In the case of dogs B and C, the amount of SNP and indels increased after the recurrence, whereas the number of changes decreased in the case of dog A.

In Supplementary Table S1, we present the most common SNP and indel positions in the analysed samples. The visual representation of the distribution of mutations, polymorphisms and heteroplasmy in the canine mitogenome is shown on the heat map in Supplementary Figure S1. Regardless of the tumour type, recurrence, or metastasis, the occurrence of m.5367C>T, m.5444T>C, m.6065A>G, m.8368C>T, m.8807G>A, m.9911_9912insTG, m.13299T>A, and m.15814T>C was confirmed in all the samples. These polymorphisms are considered to be unaffected by any molecular transformations related to carcinogenesis. Interestingly, polymorphism m.15639T>G was identified only in the samples from the crossbreed dog, which was diagnosed with *haemangiopericytoma*.

The whole mitochondrial DNA sequencing analysis allowed us to indicate new SNPs and indels which were not present in the European Variation Archive (EVA) database before: m.6743G>A, m.11457T>C, m.11457T/C (heteroplasmy), m.11998T>C, m.15955C>T, and m.16431C>T (Table 2).

We observed polymorphisms in five tRNA genes: *tRNA-Phe*, *tRNA-Leu (UUR)*, *tRNA-Trp*, *tRNA-Thr*, and *tRNA-Pro*; however, polymorphisms m.2679_2680insG, and m.2683G>A *tRNA-Leu (UUR)* were the most common (Table 3). The polymorphisms detected in these genes were primarily located in the DHU and TΨC loops and in the central loop. We observed a high prevalence of polymorphisms in *12s* and *16s rRNA* for the crossbreed dog with *haemangiopericytoma.* The exact location of these variants is indicated in Table 3. The presence of mtDNA variants was higher for the *16s rRNA* gene than for the *12s rRNA* gene.

Heteroplasmy outside the VNTR region was found in six positions: m.557A/G, m.5720G/A, m.8281T/C, m.8369C/T, m.12330A/G, and m.14977T/C. We detected the heteroplasmy outside the VNTR region in each of the three dogs (Table 4). The m.8369C/T heteroplasmy observed in the *ATP6* gene and m.12330A/G in the *ND5* gene caused nonsynonymous changes in the amino acid protein sequences—p.Pro136=/Ser and p.Thr185=/Ala, respectively, which may be interpreted as a protein heteroplasmy (Table 5).

In total, we found 21 nonsynonymous variants in the mtDNA sequence leading to amino acid sequence alterations. These variants were found in twelve out of thirteen mitochondrial protein sequences. Only the changes in the *CYTB*, *ND3*, and *ATP8* genes were always synonymous in each sample analysed. We used the Sorting Intolerant from Tolerant (SIFT) tool in order to determine the deleterious effect of the variants on the protein. The m.12346T>A and m.12636T>C (ND5) as well as m.9911_9912insTG (ND4L) variants caused deleterious effects on proteins (Table 5). Most of the nonsynonymous changes caused an amino acid change from valine to isoleucine or from methionine to threonine.

Using the Prot Param tool, we determined the effect of these nonsynonymous changes on such protein features as weight, theoretical pI, instability index, aliphatic index, and grand average of hydropathicity (GRAVY). We concluded that the nonsynonymous changes had no greater impact on either the protein stability or the aliphatic index and GRAVY. Noteworthy, the p.Phe250Leu variant found in dog B decreased the instability index value of the ND1 protein, whereas the p.Met1Val variant found in all the samples caused the transformation of instable ND4L proteins into stable ones. Details are presented in Supplementary Table S2. With the use of the SOPMA tool, we described the impact of nonsynonymous variants on protein structure, i.e., the % count of alpha helices, extended strands, β-turns, and random coils (Supplementary Table S3). Our results showed that, in most of the cases, the nonsynonymous variants slightly changed the composition of secondary structures of mitochondrial proteins. In the case of the CYTB, ND3, and ATP8 proteins, the structure was unchanged.

**Figure 2:**
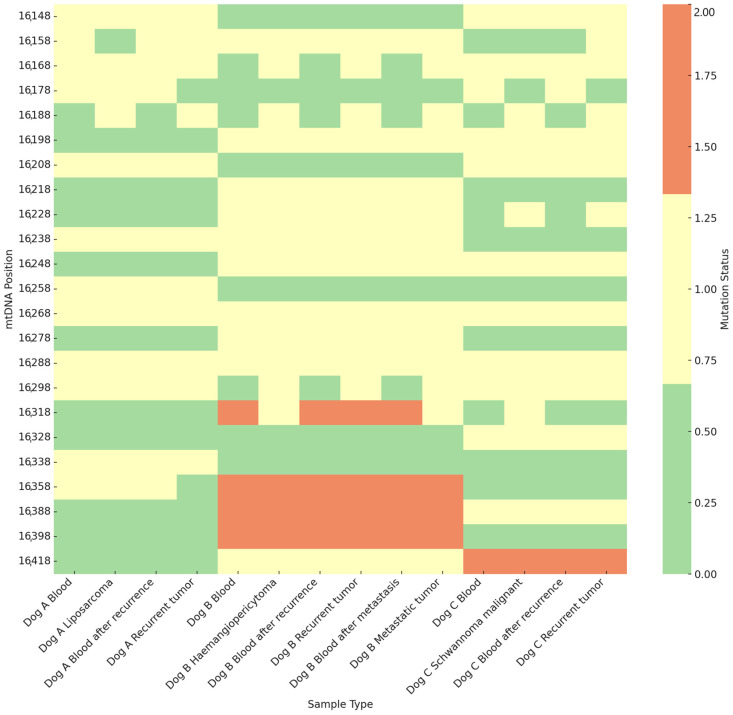
Heatmap of mtDNA variations in the VNTR region across different analysed tissues. 0—no changes observed, 1—heteroplasmy transformation from the wild to mutant type, 2—mutation.

The highest variation was observed in the VNTR region of the D-loop. In this region, three different variants were present: GTACACGTAC, GTACACGTGC, and GTACACGTA/GC. The highest heteroplasmy rate was observed for the recurrent tumour sample from dog C, whereas the lowest heteroplasmy rate was observed for the recurrent tumour sample from dog A (Figure 2). It is worth noting that the heteroplasmy shift was more often from the A to G motif than from G to A. The heatmap demonstrates hotspots within the VNTR region in positions m.16318, m.16358, m.16388, and m.16398 for dog B and in m.16418 for dog C. No mutations were observed for dog A.

The VNTR motif was identical with the reference sequence in all the analysed samples in 7 out of 30 (23%) positions: m.16138A, m.16308G, m.16348G, m.16368G, m.16378G, m.16408G, and m.16428A. Heteroplasmy in m.16268A/G and m.16288A/G was detected in all the analysed samples (2 out of 30 positions in a motif, 6%). In the m.16158A position, the heteroplasmy was detected in all the analysed samples except the primordial tumour of dog A, whereas heteroplasmy in the case of dog C was observed solely in the recurrent tumour. In all the analysed cases, the heteroplasmy in m.16188A/G was detected only in the tumour tissues. In positions m.16168A and m.16298A, the heteroplasmy was detected solely in the tumour tissues of dog B. Conversely, in position m.16178A, the heteroplasmy was observed only in the blood of dog C. The detailed description of differences in the variants in the VNTR region is presented in Supplementary Table S4.

## 3. Discussion

This study provides novel insights into the mitochondrial DNA alterations associated with recurrent and metastatic tumours in dogs. This is the first study in which the ONT was used in the analysis of molecular changes in the mtDNA of dogs with tumours. The use of the ONT in our study has highlighted the advantages of real-time long-read sequencing technologies in capturing the complete spectrum of mtDNA mutations. This approach has proven essential for understanding the complex mutational landscape of mitochondrial genomes in cancerous tissues, offering insights that surpass those provided by traditional sequencing methods [5]. It should be emphasized that the ONT is particularly important in the verification of the heteroplasmy observed in the VNTR region. Different variants of the VNTR motif and the exact percentage of observed nucleotides were identified. In our previous studies [13,14], we used well-known NGS sequencing techniques; yet, the ONT was more thorough and precise in determining the heteroplasmy level in the VNTR region. However, the challenges associated with sequencing errors, particularly in the regions of homopolymers, require cautious interpretation of the data. The general rules according to the verification of false positive results were the same as in the case of canine transmissible cancer described by Strakova et al. (2016) [15]. Such technical limitations highlight the need for continued advancements in sequencing technologies and data analysis methods to enhance the reliability of mtDNA mutation profiling [16].

Studies on the clonal expansion of tumour cells indicate that genetic drift plays a significant role in the accumulation of mtDNA variations and tumour development. Clonal expansions driven by genetic drift in different types of cancers are reported consistently, supporting the stochastic process of mtDNA mutations in tumorigenesis [17,18]. Mutations of mtDNA are considered the major process underlying mitochondrial dysfunction, especially in the context of aging and carcinogenesis. These mutations occur initially at random, but a lack of recombination and the replication advantages that some mutations may confer can lead to the proliferation of deletions and point mutations throughout the mitochondrial genome [19]. Notably, studies of mtDNA mutations linked to aging show that clonal expansions increase with age, causing abrupt transcriptional reprogramming and mitochondrial dysfunction. This is exemplified by the progressive accumulation of the mtDNA 3243A>G mutation [18,20]. Interestingly, the mtDNA mutational profile observed in colorectal cancers closely resembles that found in normal aging colonic crypts [21]. While these findings highlight the random nature of genetic drift in clonal expansions, they also suggest potential selective advantages that these mutations may provide, influencing tumour growth and adaptation. Thus, the interplay between random drift and selective pressures is crucial to understanding tumour biology and its implications for carcinogenesis.

Comparative analyses suggest that many sites in the D-loop are indeed conserved across species, including dogs. Three conserved sequence blocks in the canine mitogenome are located in regions *MT-CSB1* (positions: 16,098–16,120), *MT-CSB2* (16,461–16,477), and *MT-CSB3* (16,518–16,535). This conservation underscores the potential functional importance of these regions, as evolutionary pressures are likely to preserve sequences critical for mitochondrial function. Moreover, in the canine and human D-loop, there are several regions that are key points of gene expression and replication regulation, i.e., *MT-TAS2* (extended termination-associated sequence, positions: 15,530–15,587), *MT-3H* (mt3 H-strand control element, positions: 16,630–16,637), *MT-3L* (L-strand control element, positions 15,787–15,794), *MT-4H* (mt4 H-strand control element, positions: 15,894–15,902), and *MT-5* (control element, positions: 15,565–15,579) [22]. In our study, we did not find any mutations or variants that would have an impact on these regions. Nonetheless, the thorough analysis of mtDNA depletion frequently observed in i.e., human carcinogenesis [23,24,25,26,27,28,29] should be verified with qPCR analysis of the number of mtDNA molecules in tumour and non-tumour tissues.

The high mutation rates detected in the VNTR region of the mtDNA of recurrent tumours compared to primary tumours could be indicative of increased genetic instability, which is often associated with cancer progression [8]. Such findings are consistent with the hypothesis that mitochondrial mutations exacerbate the production of reactive oxygen species, further promoting genetic instability [3]. On the other hand, the number of changes observed in the analysed primordial and recurrent tumours did not differ notably, which may be linked with the cell viability guaranteed by energy processes in the mitochondria (Figure 1, Supplementary Table S1). It is not excluded that the process of oxidative phosphorylation must be preserved in both healthy and tumour cells, and therefore no malignant changes were observed outside the D-loop. Ziółkowska et al. (2023) observed similar changes in the VNTR region in the case of solid mammary carcinomas [14].

The heteroplasmy outside the D-loop was observed in five genes (Table 4). Interestingly, variants m.8281T/C and m.8369C/T in the case of canine transmissible cancer were first discarded by Strakova et al. (2016) due to the proximity to an indel; yet, they had substantial support and were rescued [15]. The m.8369C/T variant caused a tolerant nonsynonymous shift from proline to serine in the amino acid sequences of all the analysed dogs. The plausible protein heteroplasmy caused by this variant should be verified in further studies (Table 5). The prevalence of the heteroplasmy outside of the D-loop did not cause a probable damage to the functioning of the gene expression products. As the heteroplasmy was observed in healthy and tumour tissues, they are not likely to be a cause or an effect of carcinogenesis. The synonymous effect on the amino acid sequence may indicate that these changes are likely to be polymerase γ errors during transcription that were not previously eliminated or an effect of reactive oxygen species. As these changes were tolerated in the cells, these dogs possess two mtDNA haplotypes (wild and mutant) that are fully functional. Mitochondria are not only the targets of oxidative stress but can themselves be sources of oxidative stress [15]. The mtDNA is easily damaged by ROS resulting from a lack of histone proteins, chromatin structure, and limited repair activity [30]. It is not excluded that the result of the changes observed in the mtDNA led to a decrease in the copy number, as observed in the heart failure case of dogs with Myxomatous Mitral Valve Disease [31]. Yet, this hypothesis should be verified on a larger cohort in future research.

## 4. Materials and Methods

### 4.1. Animals

We analysed 14 mitochondrial DNA genomes in three dogs with primordial tumours and their recurrences. One dog had a metastatic tumour after 16 months and a recurrence after 28 months. Seven samples obtained from blood (*n = 7*) and tumour tissues (*n = 7*) were analysed (Table 6). We analysed the DNA extracted from postoperative cancer tissue and the blood of the examined dogs. The dogs received neither hormone therapy nor chemotherapy. The occurrence of recurrence and metastasis was assessed by histopathological examination and classified according to the WHO histological classification [32,33]. The collected samples were routinely fixed with 10% buffered formalin (pH 7.2), passed through increasing concentrations of alcoholic solutions to acetone and xylene, and embedded in paraffin blocks. For histopathological analyses, the preparations were stained with haematoxylin and eosin and examined under a light microscope coupled with a digital camera (Olympus BX43, Olympus SC100, Tokyo, Japan) in accordance with the WHO histological recommendations (International Classification of Tumours of Domestic Animals). The study was approved by the II Local Ethical Commission for animal experiments in Lublin, Poland (resolution number 6/2013).

### 4.2. Laboratory Procedures

Total DNA was isolated with NucleoSpin^®^ DNA RapidLyse (Macherey Nagel) according to the manufacturer’s protocol. For blood samples, the standard protocol for fresh and frozen samples was used. In the case of tumour samples, a protocol for challenging samples was followed, in which additional mechanical lysis on glass beads was applied. Mitochondrial DNA was amplified with two sets of primers specified in the literature [34]. As a result, 9653 bp and 9942 bp overlapping fragments were obtained. Barcoded PCR products were sequenced with the LSK-109 library construction kit (Oxford Nanopore Technologies, Oxford, UK). Briefly, PCR products purified on magnetic beads were subjected to the end repair and dA-tailing procedure, followed by sequencing adapter ligation. The library was sequenced on the R9.4.1 Flow Cell on GridIONx5 device. The details of the length and quality of PCR reads are presented in Supplementary Table S5.

Consensus sequences were created using the Medaka consensus algorithm from BAM files [16]. Reads were aligned to the NC_002008.4 reference genome (16,727 bp) using the Minimap2 algorithm [35]. Read alignment to the consensus sequences and the consensus alignment to the reference genome were also created.

For the reads aligned to the reference, VCF files with variants were prepared using the Medaka variant algorithm. In addition, a VCF file was created for the alignment of consensus sequences to the reference genome using the SAMtools mpileup algorithm. Variants from both types of VCF files were intersected using the bcftools isec algorithm to obtain VCF files with variants between the consensus and the reference, with additional information about the variant prevalence and prediction quality for a given haplotype, derived from read phases from the Medaka variant program. The VCF and .bam files generated during the analysis were read in Integrative Genomic Viewer (IGV) version 2.8.0 [36]. The variant callings in the analysed samples were determined according to the methodology used by Strakova et al. (2016) [15]. If the frequency of readings for a variant was greater than 25%, then the variant was accepted.

ONT sequencing is particularly prone to insertion and deletion errors in homopolymeric regions due to the nature of nanopore translocation dynamics. To mitigate these errors, we incorporated the following steps: 1. We employed bioinformatics tools to identify variants located within homopolymeric regions. Variants detected in these regions were flagged as potential false positives. 2. Variants identified within homopolymeric regions were excluded from downstream analyses to prevent the inclusion of artifacts. 3. We provided a comprehensive list of the most frequent indels excluded from the analysis in Supplementary Table S6. This table details the specific indels identified as homopolymer-related artifacts, ensuring transparency in our data processing. By integrating Medaka’s advanced error correction capabilities with targeted strategies to address homopolymer-induced errors, we enhanced the accuracy of our mtDNA variant analysis.

### 4.3. Bioinformatics Analyses

The probability of deleterious mutations, i.e., a functional effect of the non-synonymous protein-coding SNP, was determined using the Panther Classification System [36]. It predicts disease-causing genetic variants using position-specific evolutionary preservation. The ExPASy Server [37] was used to characterise such physicochemical parameters as the theoretical isoelectric point (pI), instability index, aliphatic index, and grand average hydropathicity (GRAVY). SOPMA was used for the calculation of the secondary structural features of antioxidant protein sequences. Trans Membrane prediction using the Hidden Markov Model (TMHMM) was used for predicting transmembrane helices based on the Hidden Markov Model [38]. In order to predict whether an amino acid substitution is deleterious, the SIFT (sorting intolerant from tolerant) algorithm was used [39,40]. The structure of tRNA molecules was predicted in the tRNAscan-SE Search Server according to the methodology proposed by Lowe and Chan [41].

The HGVS (2016) nomenclature was used for description of variants of sequences found in the DNA and proteins [42].

## 5. Conclusions

To the best of our knowledge, this is the first study to reveal variations in primordial and recurrent tissues of the mtDNA in dogs. The study revealed that the overall number of changes observed in the primordial and recurrent tumours did not differ notably; yet, the highest number of differences was observed in the VNTR region. The results revealed the m.16188G/A heteroplasmy in the case of tumour tissues, whereas in blood the variant did not differ from the reference sequence. Moreover, the heteroplasmy in tumour tissues was also noted in positions m.16168A/G and m.16298A/G in the case of the dog with *haemangiopericytoma.*

## Figures and Tables

**Table 1:** Information on the haplotypes of the studied individuals based on 100% identity with sequences stored in GenBank.

Dog	Sample—Tissue	Haplogroup	Haplotype	Accession Number from NCBI
A	B25K—blood B25G—liposarcoma B40K—blood examined after recurrence B40G—recurrent tumour	A1	A1a1	KU291092.1
B	B47K—blood B47G—haemangiopericytoma B111K—blood examined after recurrence B111G—recurrent tumour B180K—blood examined after metastasis B180G—metastatic tumour	C1	C1b1f	KU291059.1
C	B162G—schwannoma malignum	A1	A1b1a1a	KM061500.1
	B162K—blood B169K—blood examined after recurrence B169G—recurrent tumour		A1b1a2	KM061566.1

**Table 2:** The most common SNPs and indel variants in all analysed samples as well as novel SNPs and indels not observed in the European Variation Archive (EVA) database excluding the VNTR region of the D-loop.

Gene/Region	Reference Sequence	Sequence Variants	Samples
*tRNA-Leu* (*UUR*)	m.2679_2680	m.2679_2680insG	all samples
	m.2683G	m.2683G>A	
*COX1*	m.5367C	m.5367C>T	
	m.5444T	m.5444T>C	
	m.5720G	m.5720G/A †	
	m.6065A	m.6065A>G	
*COX1*	m.6743G	m.6743G>A *	
*ATP6*	m.8368C	m.8368C>T	
*COX3*	m.8807G	m.8807G>A	
*ND4L*	m.9911_9912	m.9911_9912insTG	
*ND4*	m.11457T	m.11457T>C * m.11457T/C *	B162K, B169K, B169G B162G
*ND5*	m.11998T	m.11998T>C *	all samples of dog B
	m.13299T	m.13299T>A	all samples
D-loop	m.15955C	m.15955C>T *	dog B and C samples
	m.15639T	m.15639T>A m.15639T>G	dog A and C samples dog B samples
	m.15814T	m.15814T>C	all samples
	m.16431C	m.16431C>T *	dog B samples

* Novel sequence variants not previously described in the EVA database, † Heteroplasmy.

**Table 3:** Profile of polymorphisms and heteroplasmy present in tRNA and rRNA genes in the analysed samples.

Gene	Dogs	Genomic Position	Gene Position	Variant	tRNA Region
*tRNA-Phe*	B	16	16	m.16T>C	DHU loop
*12s rRNA*	B	381	311	m.381T>A	-
		557	487	m.557A/G †	-
*16s rRNA*	C	1351	260	m.1351A>G	-
	B	1204	113	m.1204T>C	-
		1454	363	m.1454G>A	-
		1709	618	m.1709G>A	-
		1748	657	m.1748T>C	-
		1756	665	m.1756C>T	-
		2232	1141	m.2232A>G	-
*tRNA-Leu (UUR)*	A, B, C	2679_2680	8_9	m.2679_2680insG	central loop
		2683	12	m.2683G>A	DHU loop
*tRNA-Trp*	B	5009	53	m.5009C>T	TΨC loop
*tRNA-Thr*	B	15372	49	m.15372G>A	TΨC loop
*tRNA-Pro*	B	15435	112	m.15435G>A	central loop

† Heteroplasmy.

**Table 4:** List of positions in tRNA, rRNA, and protein genes where heteroplasmy was detected. Heteroplasmy was indicated when the frequency of the mutated variant exceeded > 25%.

Gene	Reference Sequence	Variant	% freq. Variant Native/Mutated	Dog
*12s rRNA*	m.557A	m.557A/G	70%/30%	B
*COX1*	m.5720G	m.5720G/A	35%/65%	A, B, C
*ATP6*	m.8281T	m.8281T/C	53%/47%	B, C
*ATP6*	m.8369C	m.8369C/T	64%/36%	A, B, C
*ND5*	m.12330A	m.12330A/G	41%/59%	B
*CYTB*	m.14977T	m.14977T/C	44%/56%	C

**Table 5:** Profile of nonsynonymous changes occurring in mitochondrial proteins of studied DNA sequences.

Gene	Gene Variant	Codon	Dogs	Amino Acid Change	SIFT
*ND1*	m.3494T>C	TTC→CTC	B	p.Phe250Leu	TLC (0.77)
*ND2*	m.4517G>A	GTT→ATT	B	p.Val202Ile	tolerated (0.59)
	m.4503A>G	AAC→AGC		p.Asn197Ser	tolerated (0.07)
*COX1*	m.6711T>A	TCT→ACT	B	p.Ser455Thr	TLC (0.07)
*COX2*	m.7593T>C	ATA→ACA	C	p.Met187Thr	TLC (0.75)
*ATP6*	m.8369C/T †	CCC→YCC	A, B, C	p.Pro136=/Ser	tolerated (0.06)
*COX3*	m.8764G>T	CTT→CTC	B	p.Ala41Ser	TLC (0.36)
	m.8807G>A	TGC→TAC	A, B, C	p.Cys55Tyr	TLC (1)
*ND4L*	m.9911_9912insTG	ATG→GTG	A, B, C	p.Met1Val	DLC (0)
*ND4*	m.11572A>C	ATC→CTC	B	p.Ile458Leu	TLC (1)
	m.11402T>C	ATC→ACC		p.Ile401Thr	TLC (0.08)
*ND5*	m.13299T>A	TCA→ACA	A, C	p.Ser508Thr	TLC (1)
	m.11959C>T	ACA→ATA	B	p.Thr61Met	TLC (0.52)
	m.11998T>C	ATA→ACA		p.Met74Thr	TLC (0.08)
	m.13299T>A	TCA→ACA		p.Ser508Thr	TLC (1)
	m.12330A/G †	ACC→RCC		p.Thr185=/Ala	TLC (1)
	m.12346T>A	CTA→CAA		p.Leu190Gln	DLC (0.01)
	m.12636T>C	TTT→CTT		p.Phe287Leu	DLC (0)
	m.12813G>A	ACC→ATC		p.Val346Ile	TLC (1)
	m.13261C>T	GTT→ATT		p.Thr495Ile	TLC (1)
*ND6*	m.13791T>C	ATT→GTT	B	p.Ile106Val	tolerated (1)

† Heteroplasmy, TLC—tolerated low confidence, DLC—deleterious low confidence.

**Table 6:** Clinical information on dogs with the analysed tumours.

Dog’s Symbol	A	B		C
Breed	Labrador	crossbreed		Amstaff
Age [years]	8	10		9
Sex	male	male		male
Cancer type	*liposarcoma*	*haemangiopericytoma*		*schwannoma malignum*
Malignancy	malignant	locally malignant		malignant
Localisation	subcostal area	ankle		buttock
Time between visits	3 months	16 months	28 months	2 months
Character of a tumour	recurrence	metastasis	recurrence	recurrence
Tumour type diagnosed	n/a	*haemangiopericytoma*		n/a
Malignancy	n/a	locally malignant		n/a
Localisation	n/a	calcaneus area	ankle	n/a

## Data Availability

The data that supports the findings of this study are available in the Supplementary Material of this article. The data obtained after ONT sequencing generated in this study is submitted to the NCBI BioProject database under accession number PRJNA1188282.

## Supplementary Materials

The following supporting information can be downloaded at: https://www.mdpi.com/article/10.3390/ijms252413414/s1.
