# Supplementary material for: Defects in the Mitochondrial Genome of Dogs with Recurrent Tumours
Source: Int J Mol Sci. 2024 Dec 14;25(24):13414. doi: 10.3390/ijms252413414 (PMC11678272; doi:10.3390/ijms252413414)
Supplement: Supplementary file 1 [file ijms-25-13414-s001.zip › ijms-3313115-supplementary.pdf]

*Supplementary Material*

# Defects in the mitochondrial genome of dogs with recurrent tumours

**Krzysztof Kowal [1], Kaja Ziółkowska-Twarowska [1], Angelika Tkaczyk-Wlizło [1], Ludmiła Grzybowska-Szatkowska [2], and Brygida Ślaska [1,*]**

[1] Institute of Biological Bases of Animal Production, University of Life Sciences in Lublin, Lublin, Akademicka 13 St., 20-950, Poland; krzysztof.kowal@up.lublin.pl, kaja.ziolkowska@up.lublin.pl, angelika.tkaczyk@up.lublin.pl

[2] Department of Radiotherapy, Medical University of Lublin, Chodźki 7, 20-093, Lublin, Poland; ludmila.grzybowska-szatkowska@umlub.pl

* Correspondence: brygida.slaska@up.lublin.pl

**Table of contents:**

**Supplementary table S1.** List of all changes detected in analysed sequences obtained from dogs with recurrent tumours generated using Unipro uGene. ........ 2

**Supplementary figure S1.** Heatmap of mtDNA variations in the whole mitogenome across different analysed tissues. 0—no changes observed, 1—heteroplasmy transformation from the wild to mutant type, 2—mutation........ 5

**Supplementary table S2.** Protein profiles based on data from Prot Param........ 6

**Supplementary table S3.** Protein profiles based on data from SOPMA. ........ 7

**Supplementary table S4.** Differences in variants in the VNTR region of the tested dogs. ........ 8

**Supplementary table S5.** Detailed information about the length and quality of PCR generated in Oxford Nanopore Technologies. ........ 9

**Supplementary table S6.** List of false positive INDELS excluded from analysis due to low frequency of reads and proximity to homopolymer regions........ 10

**Supplementary table S1.** List of all changes detected in analysed sequences obtained from dogs with recurrent tumours generated using Unipro uGene.

| DOG | | A | | | | B | | | | | | C | | | | N. of variants observed in each position |
|---|---|---|---|---|---|---|---|---|---|---|---|---|---|---|---|---|
| POSITION | SEQ. REF. | B25K | B25G | B40K | B40G | B47K | B47G | B111K | B111G | B180K | B180G | B162K | B162G | B169K | B169G | |
| 16 | T | . | . | . | . | C | C | C | C | C | C | . | . | . | . | **6** |
| 381 | T | . | . | . | . | A | A | A | A | A | A | . | . | . | . | **6** |
| 557 | A | . | . | . | . | R | R | R | R | R | R | . | . | . | . | **6** |
| 1204 | T | . | . | . | . | C | C | C | C | C | C | . | . | . | . | **6** |
| 1351 | A | . | . | . | . | . | . | . | . | . | . | G | G | G | G | **4** |
| 1439 | A | . | R | . | . | . | . | . | . | . | . | . | . | . | . | **1** |
| 1454 | G | . | . | . | . | A | A | A | A | A | A | . | . | . | . | **6** |
| 1709 | G | . | . | . | . | A | A | A | A | A | A | . | . | . | . | **6** |
| 1748 | T | . | . | . | . | C | C | C | C | C | C | . | . | . | . | **6** |
| 1756 | C | . | . | . | . | T | T | T | T | T | T | . | . | . | . | **6** |
| 2232 | A | . | . | . | . | G | G | G | G | G | G | . | . | . | . | **6** |
| 2679_2680 | - | G | G | G | G | G | G | G | G | G | G | G | G | G | G | **14** |
| 2683 | G | A | A | A | A | A | A | A | A | A | A | A | A | A | A | **14** |
| 2962 | C | . | . | . | . | T | T | T | T | T | T | T | T | T | T | **10** |
| 3196 | T | . | . | . | . | C | C | C | C | C | C | C | C | C | C | **10** |
| 3406 | C | . | . | . | . | T | T | T | T | T | T | . | . | . | . | **6** |
| 3469 | G | . | . | . | . | A | A | A | A | A | A | . | . | . | . | **6** |
| 3494 | T | . | . | . | . | C | C | C | C | C | C | . | . | . | . | **6** |
| 3598 | G | . | . | . | . | A | A | A | A | A | A | . | . | . | . | **6** |
| 4234 | C | . | . | . | . | T | T | T | T | T | T | . | . | . | . | **6** |
| 4303 | A | . | . | . | . | . | . | . | . | . | . | G | G | G | G | **4** |
| 4503 | A | . | . | . | . | G | G | G | G | G | G | . | . | . | . | **6** |
| 4517 | G | . | . | . | . | A | A | A | A | A | A | . | . | . | . | **6** |
| 4940 | T | . | . | . | . | . | . | . | . | . | . | C | C | C | C | **4** |
| 5009 | C | . | . | . | . | T | T | T | T | T | T | . | . | . | . | **6** |
| 5367 | C | T | T | T | T | T | T | T | T | T | T | T | T | T | T | **14** |
| 5444 | T | C | C | C | C | C | C | C | C | C | C | C | C | C | C | **14** |
| 5624 | G | . | . | . | . | A | A | A | A | A | A | . | . | . | . | **6** |
| 5720 | G | R | R | R | R | R | R | R | R | R | R | R | R | R | R | **14** |
| 6065 | A | G | G | G | G | G | G | G | G | G | G | G | G | G | G | **14** |
| 6257 | G | . | . | . | . | A | A | A | A | A | A | . | . | . | . | **6** |
| 6401 | C | . | . | . | . | . | . | . | . | . | . | T | T | T | T | **4** |
| 6470 | G | . | . | . | . | A | A | A | A | A | A | . | . | . | . | **6** |
| 6554 | T | . | . | . | . | . | . | . | . | . | . | C | C | C | C | **4** |
| 6629 | T | C | C | C | C | . | . | . | . | . | . | . | . | . | . | **4** |
| 6711 | T | . | . | . | . | A | A | A | A | A | A | . | . | . | . | **6** |
| 6740 | G | . | . | . | . | A | A | A | A | A | A | . | . | . | . | **6** |
| 6743 | G | . | . | . | . | A | A | A | A | A | A | . | . | . | . | **6** |
| 6863 | C | . | . | . | . | T | T | T | T | T | T | . | . | . | . | **6** |
| 7058 | T | . | . | . | . | C | C | C | C | C | C | . | . | . | . | **6** |
| 7593 | T | . | . | . | . | . | . | . | . | . | . | C | C | C | C | **4** |
| 8221 | A | . | . | . | . | C | C | C | C | C | C | . | . | . | . | **6** |
| 8225 | T | . | . | . | . | C | C | C | C | C | C | . | . | . | . | **6** |
| 8281 | T | . | . | . | . | Y | Y | Y | Y | Y | Y | Y | Y | Y | Y | **10** |
| 8323 | A | . | . | . | . | G | G | G | G | G | G | . | . | . | . | **6** |
| 8368 | C | T | T | T | T | T | T | T | T | T | T | T | T | T | T | **14** |
| 8369 | C | Y | Y | Y | Y | Y | Y | Y | Y | Y | Y | Y | Y | Y | Y | **14** |
| 8425 | G | . | . | . | . | A | A | A | A | A | A | . | . | . | . | **6** |
| 8536 | C | T | T | T | T | . | . | . | . | . | . | . | . | . | . | **4** |
| 8703 | G | . | . | . | . | A | A | A | A | A | A | . | . | . | . | **6** |
| 8760 | A | . | . | . | . | G | G | G | G | G | G | . | . | . | . | **6** |
| 8764 | G | . | . | . | . | T | T | T | T | T | T | . | . | . | . | **6** |
| 8807 | G | A | A | A | A | A | A | A | A | A | A | A | A | A | A | **14** |

| *cont.* | | | | | | | | | | | | | | | | |
|---|---|---|---|---|---|---|---|---|---|---|---|---|---|---|---|---|
| 8877 | A | . | . | . | . | G | G | G | G | G | G | . | . | . | . | **6** |
| 8991 | A | . | . | . | . | G | G | G | G | G | G | . | . | . | . | **6** |
| 9222 | C | . | . | . | . | T | T | T | T | T | T | . | . | . | . | **6** |
| 9708 | C | . | . | . | . | T | T | T | T | T | T | . | . | . | . | **6** |
| 9911_9912 | - | TG | TG | TG | TG | TG | TG | TG | TG | TG | TG | TG | TG | TG | TG | **14** |
| 10165 | C | T | T | T | T | . | . | . | . | . | . | . | . | . | . | **4** |
| 10263 | C | . | . | . | . | T | T | T | T | T | T | . | . | . | . | **6** |
| 10404 | C | . | . | . | . | T | T | T | T | T | T | . | . | . | . | **6** |
| 10533 | A | . | . | . | . | T | T | T | T | T | T | . | . | . | . | **6** |
| 10611 | A | . | . | . | . | . | . | . | . | . | . | T | T | T | T | **4** |
| 10776 | T | . | . | . | . | C | C | C | C | C | C | . | . | . | . | **6** |
| 10785 | A | . | . | . | . | G | G | G | G | G | G | . | . | . | . | **6** |
| 10917 | G | . | . | . | . | A | A | A | A | A | A | . | . | . | . | **6** |
| 10992 | G | . | . | . | . | A | A | A | A | A | A | A | A | A | A | **10** |
| 11247 | A | . | . | . | . | G | G | G | G | G | G | . | . | . | . | **6** |
| 11250 | T | . | . | . | . | C | C | C | C | C | C | . | . | . | . | **6** |
| 11322 | T | . | . | . | . | C | C | C | C | C | C | . | . | . | . | **6** |
| 11400 | T | . | . | . | . | C | C | C | C | C | C | . | . | . | . | **6** |
| 11402 | T | . | . | . | . | C | C | C | C | C | C | . | . | . | . | **6** |
| 11457 | T | . | . | . | . | . | . | . | . | . | . | C | Y | C | C | **4** |
| 11572 | A | . | . | . | . | C | C | C | C | C | C | . | . | . | . | **6** |
| 11959 | C | . | . | . | . | T | T | T | T | T | T | . | . | . | . | **6** |
| 11963 | C | . | . | . | . | T | T | T | T | T | T | . | . | . | . | **6** |
| 11998 | T | . | . | . | . | C | C | C | C | C | C | . | . | . | . | **6** |
| 12122 | C | . | . | . | . | T | T | T | T | T | T | . | . | . | . | **6** |
| 12200 | C | . | . | . | . | . | . | . | . | . | . | T | T | T | T | **4** |
| 12272 | T | . | . | . | . | C | C | C | C | C | C | . | . | . | . | **6** |
| 12330 | A | . | . | . | . | R | R | R | R | R | R | . | . | . | . | **6** |
| 12346 | T | . | . | . | . | A | A | A | A | A | A | . | . | . | . | **6** |
| 12636 | T | . | . | . | . | C | C | C | C | C | C | . | . | . | . | **6** |
| 12788 | T | . | . | . | . | C | C | C | C | C | C | . | . | . | . | **6** |
| 12813 | G | . | . | . | . | A | A | A | A | A | A | . | . | . | . | **6** |
| 13261 | C | . | . | . | . | T | T | T | T | T | T | . | . | . | . | **6** |
| 13299 | T | A | A | A | A | A | A | A | A | A | A | A | A | A | A | **14** |
| 13319 | C | . | . | . | . | T | T | T | T | T | T | . | . | . | . | **6** |
| 13618 | A | . | . | . | . | G | G | G | G | G | G | . | . | . | . | **6** |
| 13660 | C | . | . | . | . | T | T | T | T | T | T | . | . | . | . | **6** |
| 13708 | C | . | . | . | . | T | T | T | T | T | T | . | . | . | . | **6** |
| 13777 | G | . | . | . | . | A | A | A | A | A | A | . | . | . | . | **6** |
| 13791 | T | . | . | . | . | C | C | C | C | C | C | . | . | . | . | **6** |
| 14608 | A | . | . | . | . | G | G | G | G | G | G | . | . | . | . | **6** |
| 14647 | T | . | . | . | . | C | C | C | C | C | C | . | . | . | . | **6** |
| 14692 | G | . | . | . | . | A | A | A | A | A | A | . | . | . | . | **6** |
| 14977 | T | . | . | . | . | . | . | . | . | . | . | Y | Y | Y | Y | **4** |
| 15185 | T | . | . | . | . | C | C | C | C | C | C | . | . | . | . | **6** |
| 15214 | G | . | . | . | . | A | A | A | A | A | A | A | A | A | A | **10** |
| 15372 | G | . | . | . | . | A | A | A | A | A | A | . | . | . | . | **6** |
| 15435 | G | . | . | . | . | A | A | A | A | A | A | . | . | . | . | **6** |
| 15508 | C | . | . | . | . | T | T | T | T | T | T | . | . | . | . | **6** |
| 15526 | C | . | . | . | . | T | T | T | T | T | T | . | . | . | . | **6** |
| 15611 | T | . | . | . | . | C | C | C | C | C | C | . | . | . | . | **6** |
| 15620 | T | . | . | . | . | . | . | . | . | . | . | C | C | C | C | **4** |
| 15627 | A | . | . | . | . | . | . | . | . | . | . | G | G | G | G | **4** |
| 15639 | T | A | A | A | A | G | G | G | G | G | G | A | A | A | A | **14** |
| 15650 | T | . | . | . | . | C | C | C | C | C | C | . | . | . | . | **6** |
| 15710 | C | . | . | . | . | T | T | T | T | T | T | . | . | . | . | **6** |
| 15800 | T | . | . | . | . | C | C | C | C | C | C | . | . | . | . | **6** |
| 15814 | C | T | T | T | T | T | T | T | T | T | T | T | T | T | T | **14** |
| 15912 | C | . | . | . | . | T | T | T | T | T | T | . | . | . | . | **6** |

| *cont.* | | | | | | | | | | | | | | | | |
|---|---|---|---|---|---|---|---|---|---|---|---|---|---|---|---|---|
| 15955 | C | . | . | . | . | T | T | T | T | T | T | T | T | T | T | **10** |
| 16003 | A | . | . | . | . | G | G | G | G | G | G | . | . | . | . | **6** |
| 16025 | T | C | C | C | C | . | . | . | . | . | . | . | . | . | . | **4** |
| 16148 | A | R | R | R | R | . | . | . | . | . | . | R | R | R | R | **8** |
| 16158 | A | R | . | R | R | R | R | R | R | R | R | . | . | . | R | **10** |
| 16168 | A | R | R | R | R | . | R | . | R | . | R | R | R | R | R | **11** |
| 16178 | A | R | R | R | . | . | . | . | . | . | . | R | . | R | . | **5** |
| 16188 | G | . | R | . | R | . | R | . | R | . | R | . | R | . | R | **7** |
| 16198 | G | . | . | . | . | R | R | R | R | R | R | R | R | R | R | **10** |
| 16208 | A | R | R | R | R | . | . | . | . | . | . | R | R | R | R | **8** |
| 16218 | G | . | . | . | . | R | R | R | R | R | R | . | . | . | . | **6** |
| 16228 | G | . | . | . | . | R | R | R | R | R | R | . | R | . | R | **8** |
| 16238 | G | R | R | R | R | R | R | R | R | R | R | . | . | . | . | **10** |
| 16248 | A | . | . | . | . | R | R | R | R | R | R | R | R | R | R | **10** |
| 16258 | G | R | R | R | R | . | . | . | . | . | . | . | . | . | . | **4** |
| 16268 | A | R | R | R | R | R | R | R | R | R | R | R | R | R | R | **14** |
| 16278 | G | . | . | . | . | R | R | R | R | R | R | . | . | . | . | **6** |
| 16288 | A | R | R | R | R | R | R | R | R | R | R | R | R | R | R | **14** |
| 16298 | A | R | R | R | R | . | R | . | R | . | R | R | R | R | R | **11** |
| 16318 | A | . | . | . | . | G | R | G | G | G | R | . | R | . | . | **7** |
| 16328 | G | . | . | . | . | . | . | . | . | . | . | R | R | R | R | **4** |
| 16338 | G | R | R | R | R | . | . | . | . | . | . | . | . | . | . | **4** |
| 16358 | A | R | R | R | . | G | G | G | G | G | G | . | . | . | . | **9** |
| 16388 | A | . | . | . | . | G | G | G | G | G | G | R | R | R | R | **10** |
| 16398 | A | . | . | . | . | G | G | G | G | G | G | . | . | . | . | **6** |
| 16418 | A | . | . | . | . | R | R | R | R | R | R | G | G | G | G | **10** |
| 16431 | C | . | . | . | . | T | T | T | T | T | T | . | . | . | . | **6** |
| 16439 | T | . | . | . | . | C | C | C | C | C | C | . | . | . | . | **6** |
| 16664 | T | . | . | . | . | . | . | . | . | . | . | Y | Y | Y | Y | **4** |
| 16665 | T | . | . | . | . | . | . | . | . | . | . | Y | Y | Y | Y | **4** |
| 16671 | T | . | . | . | . | Y | Y | Y | Y | Y | Y | . | . | . | . | **6** |
| 16672 | C | . | . | . | . | . | . | . | . | . | . | Y | Y | Y | Y | **4** |
| **N. of changes observed in each sample** | | **90** | **91** | **90** | **89** | **176** | **179** | **176** | **179** | **176** | **179** | **107** | **109** | **107** | **109** | |

**Supplementary figure S1.** Heatmap of mtDNA variations in the whole mitogenome across different analysed tissues. 0—no changes observed, 1—heteroplasmy transformation from the wild to mutant type, 2—mutation.

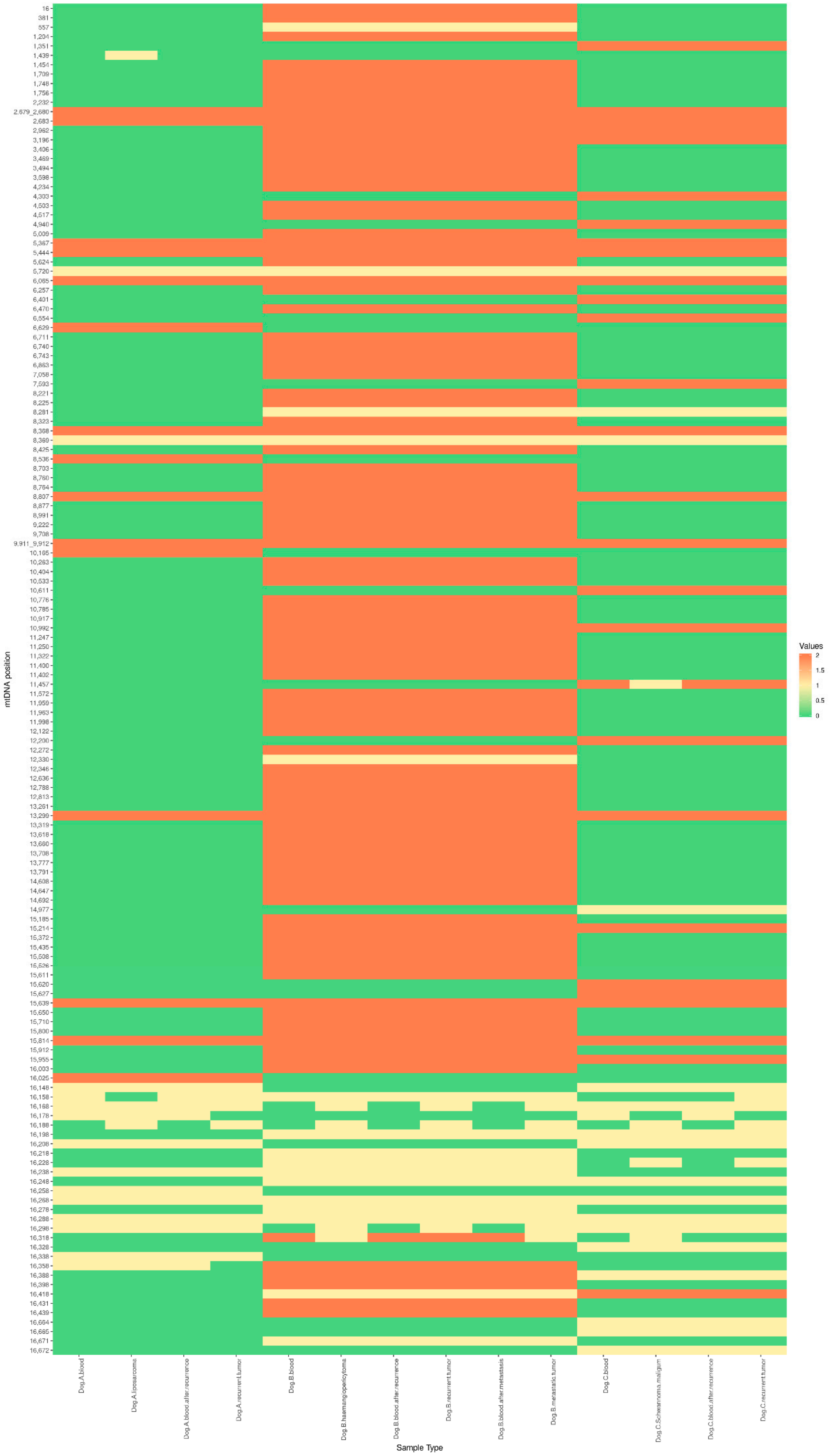

**Supplementary table S2.** Protein profiles based on data from Prot Param.

| Protein | Dogs | Amino acid change | Number of amino acids | Weight | Theoretical pI (Isoelectric point) | Instability index above >40 - unstable | Aliphatic index | Grand average of hydropathicity (GRAVY) |
|---|---|---|---|---|---|---|---|---|
| **ND1** | **A, C** | **No changes** | 318 | 35892,30 | 7,77 | **46,15** | 126,45 | 0,859 |
| | **B** | p.Phe250Leu | 318 | 35858,28 | 7,77 | **45,55** | 127,67 | 0,862 |
| **ND2** | **A, C** | **No changes** | 347 | 39101,88 | 9,86 | 29,67 | 120,61 | 0,762 |
| | **B** | p.Asn197Ser | 347 | 39088,89 | 9,86 | 31,58 | 120,89 | 0,77 |
| | | p.Val202Ile | | | | | | |
| **COX1** | **A, C** | **No changes** | 514 | 57039,28 | 6,10 | 27,96 | 101,89 | 0,687 |
| | **B** | p.Ser455Thr | 514 | 57053,31 | 6,10 | 27,59 | 101,89 | 0,678 |
| **COX2** | **A, B** | **No changes** | 227 | 26061,55 | 4,62 | 36,64 | 109,91 | 0,296 |
| | **C** | p.Met187Thr | 227 | 26031,46 | 4,62 | 36,97 | 109,91 | 0,285 |
| **ATP8** | **A, B, C** | **No changes** | 67 | 7978,38 | 9,16 | 36,04 | 84,48 | -0,1 |
| **ATP6** | **—** | **No changes** | 226 | 24789,02 | 10,11 | 32,34 | 140,75 | 0,977 |
| | **A, B, C** | p.Pro136=/Ser † | 226 | 24778,98 | 10,11 | 31,49 | 140,75 | 0,981 |
| **COX3** | **—** | **No changes** | 261 | 29874,66 | 6,44 | 19,67 | 93,37 | 0,459 |
| | **B** | p.Ala41Ser | 261 | 29950,7 | 6,44 | 22,08 | 92,99 | 0,434 |
| | | p.Cys55Tyr | | | | | | |
| | **A, C** | p.Cys55Tyr | 261 | 29934,7 | 6,44 | 20,87 | 93,37 | 0,444 |
| **ND3** | **A, B, C** | **No changes** | 115 | 13009,62 | 4,44 | 30,24 | 127,3 | 0,798 |
| **ND4L** | **—** | **No changes** | 98 | 10862,32 | 5,27 | **43,22** | 140,2 | 1,302 |
| | **A, B, C** | p.Met1Val | 98 | 10830,26 | 5,27 | 38,74 | 143,16 | 1,326 |
| **ND4** | **A, C** | **No changes** | 459 | 52095,21 | 9,58 | 37,22 | 131,37 | 0,801 |
| | **B** | p.Ile401Thr | 459 | 52083,15 | 9,58 | 37,22 | 130,52 | 0,788 |
| | | p.Ile458Leu | | | | | | |
| **ND5** | **—** | **No changes** | 606 | 68361,4 | 9,28 | 31,51 | 109,95 | 0,591 |
| | **A, C** | p.Ser508Thr | 606 | 68375,43 | 9,28 | 31,07 | 109,95 | 0,591 |
| | **B** | p.Thr61Met | 606 | 68352,44 | 9,28 | 31,64 | 110,92 | 0,594 |
| | | p.Met74Thr | | | | | | |
| | | p.Thr185=/Ala † | | | | | | |
| | | p.Leu190Gln | | | | | | |
| | | p.Phe287Leu | | | | | | |
| | | p.Val346Ile | | | | | | |
| | | p.Thr495Ile | | | | | | |
| | | p.Ser508Thr | | | | | | |
| **ND6** | **A, C** | **No changes** | 175 | 18825,27 | 4,11 | 21,49 | 122,97 | 1,054 |
| | **B** | p.Ile106Val | 175 | 18811,24 | 4,11 | 20,39 | 122,4 | 1,052 |
| **CYTB** | **A, B, C** | **No changes** | 379 | 42593,51 | 6,85 | 38,39 | 120,95 | 0,715 |

**† Heteroplasmy, no changes** means that the samples were identical to the reference sequence or identified variants did not change the amino acid sequence.

**Supplementary table S3.** Protein profiles based on data from SOPMA.

| Protein | Dogs | Amino acid change | Alpha helix | Extended strand | Beta turn | Random coil |
|---|---|---|---|---|---|---|
| **ND1** | **A,C** | **No changes** | **56,29%** | **14,47%** | **3,46%** | **25,79%** |
| | **B** | p.Phe250Leu | 56,29% | 12,26% | 1,57% | 29,87% |
| **ND2** | **A,C** | **No changes** | **53,60%** | **15,27%** | **3,46%** | **27,67%** |
| | **B** | p.Asn197Ser<br>p.Val202Ile | 53,31% | 15,56% | 3,75% | 27,38% |
| **COX1** | **A,C** | **No changes** | **51,95%** | **14,98%** | **6,03%** | **27,04%** |
| | **B** | p.Ser455Thr | 49,22% | 15,56% | 6,81% | 28,40% |
| **COX2** | **A,B** | **No changes** | **31,72%** | **24,23%** | **3,08%** | **40,97%** |
| | **C** | p.Met187Thr | 29,07% | 23,35% | 3,96% | 43,61% |
| **ATP8** | **A,B,C** | **No changes** | **19,40%** | **11,94%** | | **68,66%** |
| **ATP6** | **–** | **No changes** | **65,04%** | **6,19%** | **1,33%** | **27,43%** |
| | **A,B,C** | p.Pro136=/Ser † | 66,37% | 6,64% | 0,44% | 26,55% |
| **COX3** | **–** | **No changes** | **53,26%** | **14,18%** | **4,60%** | **27,97%** |
| | **B** | p.Ala41Ser<br>p.Cys55Tyr | 50,57% | 13,79% | 5,36% | 30,27% |
| | **A, C** | p.Cys55Tyr | 53,64% | 14,18% | 4,98% | 27,20% |
| **ND3** | **A, B** | **No changes** | **60,87%** | **3,48%** | **2,61%** | **33,04%** |
| | **C** | p.Glu115Lys | 66,09% | 2,61% | 0,87% | 30,43% |
| **ND4L** | **–** | **No changes** | **83,67%** | **6,12%** | **2,04%** | **8,16%** |
| | **A,B,C** | p.Met1Val | 72,45% | 8,16% | 2,04% | 17,35% |
| **ND4** | **A,C** | **No changes** | **50,98%** | **15,90%** | **3,49%** | **29,63%** |
| | **B** | p.Ile401Thr<br>p.Ile458Leu | 50,11% | 16,34% | 4,14% | 29,41% |
| **ND5** | **–** | **No changes** | **55,61%** | **12,87%** | **2,31%** | **29,21%** |
| | **A,C** | p.Ser508Thr | 56,27% | 13,20% | 2,31% | 28,22% |
| | **B** | p.Thr61Met<br>p.Met74Thr<br>p.Thr185=/Ala †<br>p.Leu190Gln<br>p.Phe287Leu<br>p.Val346Ile<br>p.Thr495Ile<br>p.Ser508Thr | 58,25% | 11,55% | 1,65% | 28,55% |
| **ND6** | **A,C** | **No changes** | **40,00%** | **29,14%** | **7,43%** | **23,43%** |
| | **B** | p.Ile106Val | 41,71% | 29,71% | 6,86% | 21,71% |
| **CYTB** | **A,B,C** | **No changes** | 53,03% | 9,23% | 3,17% | 34,56% |

**† Heteroplasmy, no changes** means that the samples were identical to the reference sequence or identified variants did not change the amino acid sequence.

**Supplementary table S4.** Differences in variants in the VNTR region of the tested dogs.

| **DOG** | **A** | | | | **B** | | | | | | **C** | | | |
|---|---|---|---|---|---|---|---|---|---|---|---|---|---|---|
| **SEQ. REF. VARIANT** | **B25 K** | **B25 G** | **B40 K** | **B40 G** | **B47 K** | **B47 G** | **B111 K** | **B111 G** | **B180 K** | **B180 G** | **B162 K** | **B162 G** | **B169 K** | **B169 G** |
| **m.16138A** | Identical with the reference sequence in all analysed samples | | | | | | | | | | | | | |
| **m.16148A*** | m.16148A/G | | | | Identical with the ref. seq. | | | | | | m.16148A/G | | | |
| **m.16158A*** | R | A | R | R | m.16158A/G | | | | | | A | A | A | R |
| **m.16168A*** | m.16168A/G | | | | A | R | A | R | A | R | m.16168A/G | | | |
| **m.16178A*** | R | R | R | A | m.16178A/G | | | | | | R | A | R | A |
| **m.16188G*** | G | R | G | R | G | R | G | R | G | R | G | R | G | R |
| **m.16198G*** | Identical with the ref. seq. | | | | m.16198G/A | | | | | | | | | |
| **m.16208A*** | m.16208A/G | | | | Identical with the ref. seq. | | | | | | m.16208A/G | | | |
| **m.16218G*** | Identical with the ref. seq. | | | | m.16218G/A | | | | | | Identical with the ref. seq. | | | |
| **m.16228G*** | Identical with the ref. seq. | | | | m.16228G/A | | | | | | G | R | G | R |
| **m.16238G*** | m.16238G/A | | | | | | | | | | Identical with the ref. seq. | | | |
| **m.16248A*** | Identical with the ref. seq. | | | | m.16248A/G | | | | | | | | | |
| **m.16258G*** | m.16258G/A | | | | Identical with the ref. seq. | | | | | | | | | |
| **m.16268A*** | Heteroplasmy m.16268A/G in all analysed samples | | | | | | | | | | | | | |
| **m.16278G*** | Identical with the ref. seq. | | | | m.16278G/A | | | | | | Identical with the ref. seq. | | | |
| **m.16288A*** | Heteroplasmy m.16288A/G in all analysed samples | | | | | | | | | | | | | |
| **m.16298A*** | m.16298A/G | | | | A | R | A | R | A | R | m.16298A/G | | | |
| **m.16308G** | Identical with the reference sequence in all analysed samples | | | | | | | | | | | | | |
| **m.16318A*** | Identical with the ref. seq. | | | | G | R | G | G | G | R | A | R | A | A |
| **m.16328G*** | Identical with the ref. seq. | | | | | | | | | | m.16328G/A | | | |
| **m.16338G*** | m.16338G/A | | | | Identical with the ref. seq. | | | | | | | | | |
| **m.16348G** | Identical with the reference sequence in all analysed samples | | | | | | | | | | | | | |
| **m.16358A*** | R | R | R | A | m.16358A>G | | | | | | Identical with the ref. seq. | | | |
| **m.16368G** | Identical with the reference sequence in all analysed samples | | | | | | | | | | | | | |
| **m.16378G** | Identical with the reference sequence in all analysed samples | | | | | | | | | | | | | |
| **m.16388A*** | Identical with the ref. seq. | | | | m.16388A>G | | | | | | m.16388A/G | | | |
| **m.16398A** | Identical with the ref. seq. | | | | m.16389A>G | | | | | | Identical with the ref. seq. | | | |
| **m.16408G** | Identical with the reference sequence in all analysed samples | | | | | | | | | | | | | |
| **m.16418A*** | Identical with the ref. seq. | | | | m.16418A/G | | | | | | m.16418A>G | | | |
| **m.16428A** | Identical with the reference sequence in all analysed samples | | | | | | | | | | | | | |

**R – heteroplasmy A/G, * - positions with heteroplasmic variants not previously described in the European Variation Archive (EVA) database**

**Supplementary table S5.** Detailed information about the length and quality of PCR generated in Oxford Nanopore Technologies.

| Dog | Sample | Mean read length | Mean read quality | Median read length | Median read quality | Number of reads | Read length N50 | Total bases |
|---|---|---|---|---|---|---|---|---|
| A | **B25G** | 9 764,5 | 11,9 | 9 862,0 | 12,1 | 2 391 | 9 867 | 23 346 953 |
| | **B25K** | 9 613,8 | 11,8 | 9 753,0 | 12,0 | 4 238 | 9 777 | 40 743 271 |
| | **B40G** | 9 655,0 | 11,9 | 9 830,0 | 12,0 | 5 464 | 9 837 | 52 754 837 |
| | **B40K** | 9 631,9 | 11,9 | 9 733,0 | 12,0 | 6 107 | 9 751 | 58 821 941 |
| B | **B47K** | 9 677,7 | 11,9 | 9 808,0 | 12,0 | 5 074 | 9 821 | 49 104 728 |
| | **B47G** | 9 599,5 | 11,9 | 9 640,0 | 12,1 | 7 796 | 9 643 | 74 837 685 |
| | **B111K** | 9 611,1 | 11,9 | 9 632,0 | 12,0 | 13 236 | 9 634 | 127 213 156 |
| | **B111G** | 9 592,2 | 11,8 | 9 642,5 | 12,0 | 6 110 | 9 647 | 58 608 259 |
| | **B180K** | 9 704,2 | 11,9 | 9 754,0 | 12,0 | 13 716 | 9 768 | 133 103 150 |
| | **B180G** | 9 652,4 | 11,9 | 9 650,0 | 12,1 | 11 236 | 9 654 | 108 454 484 |
| C | **B162K** | 9 661,8 | 11,9 | 9 757,0 | 12,0 | 8 417 | 9 774 | 81 322 988 |
| | **B162G** | 9 603,1 | 11,8 | 9 725,0 | 11,9 | 3 419 | 9 750 | 32 832 875 |
| | **B169K** | 9 665,2 | 11,9 | 9 725,0 | 12,0 | 9 909 | 9 737 | 95 772 738 |
| | **B169G** | 9 558,8 | 11,9 | 9 634,0 | 12,0 | 5 270 | 9 641 | 50 374 771 |

**Supplementary table S6.** List of false positive INDELS excluded from analysis due to low frequency of reads and proximity to homopolymer regions.

| *GENE/ REGION* | **VARIANT** | **N. of samples** |
|---|---|---|
| *16s rRNA* | m.1092_1093insA | **13** |
| | m.1182_1183insT | **2** |
| | m.1460_1461insT | **1** |
| | m.1753_1754insC | **1** |
| | m.2071_2072insG | **2** |
| | m.2439_2440insC | **1** |
| | m.2463_2464insG | **1** |
| | m.2549_2550insC | **1** |
| | m.2628_2629insC | **1** |
| *tRNA-Leu (UUR)* | m.2743_2744insA | **4** |
| *tRNA-Ile* | m.3717_3718insA | **14** |
| *ND2* | m.4872_4873insA | **8** |
| | m.4883_4884insC | **1** |
| | m.4886delT | **5** |
| *tRNA-Asn* | m.5135_5136insT | **2** |
| rep_origin | m.5196_5197insA | **1** |
| | m.5215_5216insC | **1** |
| *COX1* | m.6023_6024insG | **1** |
| | m.6068_6069insC | **2** |
| | m.6625_6626insC | **3** |
| | m.6753_6754insT | **1** |
| *COX2* | m.7073_7074insC | **5** |
| | m.7760_7761insT | **2** |
| *ATP6* | m.7994_7995insC | **1** |
| *COX3* | m.8896_8897insT | **13** |
| | m.8916_8917insT | **10** |
| | m.9197_9198insT | **4** |
| | m.9231_9232insT | **6** |
| | m.9299_9300insC | **1** |
| *ND3* | m.9657_9658insT | **3** |
| *ND4L* | m.9860_9861insA | **6** |

| *GENE/ REGION* | **VARIANT** | **N. of samples** |
|---|---|---|
| *ND4* | m.10421_10422insCGG | **1** |
| | m.10608_10609insG | **1** |
| *ND5* | m.12617_12618insG | **4** |
| | m.12671_12672insA | **3** |
| | m.12776_12777insT | **4** |
| | m.12875_12876insC | **2** |
| | m.13026_13027insC | **2** |
| | m.13079_13080insC | **1** |
| | m.13089_13090insC | **12** |
| | m.13123_13124insC | **1** |
| *ND6* | m.13963_13964insC | **1** |
| | m.14032_14033insG | **2** |
| *CYTB* | m.14293_14294insG | **1** |
| | m.14868_14869insC | **8** |
| | m.14875_14876insG | **2** |
| | m.14876_14877insC | **6** |
| | m.14877_14878insC | **1** |
| | m.14951_14952insC | **1** |
| | m.14972_14973insC | **2** |
| | m.15099_15100insC | **1** |
| D-loop | m.15530_15531insC | **3** |
| | m.15756_15757insG | **1** |
| | m.15931_15932insG | **1** |
| | m.15931delG | **3** |
| | m.16018_16019insC | **2** |
| | m.16130delT | **1** |
| | m.16470delT | **8** |
| | m.16563_16564insG | **4** |
| | m.16660_16661insC | **1** |
| | m.16663delT | **5** |
